# Current Awareness on Comparative and Functional Genomics

**DOI:** 10.1002/cfg.227

**Published:** 2003-04


**Comparative and Functional Genomics**
*Comp Funct Genom* 2003; 4: 277–284
Published online in Wiley InterScience (www.interscience.wiley.com). **DOI** 10.1002/cfg.227

### Current awareness on comparative and functional genomics

### 1 Reviews & symposia

2002. Special issue: Papers presented at the IIIrd Anton Dohm Workshop 'Fish Genomics: Structural and Functional Aspects' - Ischia (Naples), Italy - 1-2 June 2001. *Gene* **295:** (2)

2002. Special issue: Proceedings of the European Conference on Computational Biology (ECCB 2002). *Bioinformatics* **18:** (Suppl 2)

Adriaenssens E, Lemoine J, El Yazidi-Belkoura I, Hondermarck H. 2002. Growth signaling in breast cancer cells: Outcomes and promises of proteomics. *Biochem Pharmacol* **64:** (5-6) 797.

Arber W. 2002. Roots, strategies and prospects of functional genomics. *Curr Sci* **83:** (7) 826.

Bailey SN, Wu RZ, Sabatini DM. 2002. Applications of transfected cell microarrays in high-throughput drug discovery (Review). *Drug Discov Today* **7:** (18) S113.

Basile VS, Masellis M, Potkin SG, Kennedy JL. 2002. Pharmacogenomics in schizophrenia: The quest for individualized therapy (Review). *Hum Mol Genet* **11:** (20) 2517.

Beske OE, Goldbard S. 2002. High-throughput cell analysis using multiplexed array technologies (Review). *Drug Discov Today* **7:** (18) S131.

Birney E, Clamp M, Hubbard T. 2002. Databases and tools for browsing genomes. *Annu Rev Genomic Hum Genet* **3:** 293.

Brazhnik P, De la Fuente A, Mendes P. 2002. Gene networks: How to put the function in genomics (Review). *Trends Biotechnol* **20:** (11) 467.

Brunner D, Nestler E, Leahy E. 2002. In need of high-throughput behavioral systems. *Drug Discov Today* **7:** (18) S107.

Carson JP, Thaller C, Eichele G. 2002. A transcriptome atlas of the mouse brain at cellular resolution (Review). *Curr Opin Neurobiol* **12:** (5) 562.

Chalmers DT, Behan DP. 2002. The use of constitutively active GPCRs in drug discovery and functional genomics (Review). *Nat Rev Drug Discov* **1:** (8) 599.

Chaurand P, Caprioli RM. 2002. Direct profiling and imaging of peptides and proteins from mammalian cells and tissue sections by mass spectrometry (Review). *Electrophoresis* **23:** (18) 3125.

Dahl SG, Kristiansen K, Sylte I. 2002. Bioinformatics: From genome to drug targets. *Ann Med* **34:** (4) 306.

Dewey TG. 2002. From microarrays to networks: Mining expression time series. *Drug Discov Today* **7:** (20) S170.

Dierick JF, Eliaers F, Remacle J, Raes M, Fey SJ, Larsen PM, Toussaint O. 2002. Stress-induced premature senescence and replicative senescence are different phenotypes, proteomic evidence. *Biochem Pharmacol* **64:** (5-6) 1011.

Edwards AM, Kus B, Jansen R, Greenbaum D, Greenblatt J, Gerstein M. 2002. Bridging structural biology and genomics: Assessing protein interaction data with known complexes (Review). *Trends Genet* **18:** (10) 529.

Famulok M, Verma S. 2002. *In vivo*-applied functional RNAs as tools in proteomics and genomics research (Review). *Trends Biotechnol* **20:** (11) 462.

Gromov PS, Ostergaard M, Gromova I, Celis JE. 2002. Human proteomic databases: A powerful resource for functional genomics in health and disease (Review). *Prog Biophys Mol Biol* **80:** (1-2) 3.

Hayduk EJ, Choe LH, Lee KH. 2002. Proteomic tools in discovery-driven science. *Curr Sci* **83:** (7) 840.

Hemmila IA, Hurskainen P. 2002. Novel detection strategies for drug discovery (Review). *Drug Discov Today* **7:** (18) S150.

Hubbard MJ. 2002. Functional proteomics: The goalposts are moving. *Proteomics* **2:** (9) 1069.

Huels C, Muellner S, Meyer HE, Cahill DJ. 2002. The impact of protein biochips and microarrays on the drug development process (Review). *Drug Discov Today* **7:** (18) S119.

Ilag LL, Ng JH, Beste G, Henning SW. 2002. Emerging high-throughput drug target validation technologies (Review). *Drug Discov Today* **7:** (18) S136.

Ivanova AV, Ivanov SV. 2002. Differential display analysis of gene expression in yeast (Review). *Cell Mol Life Sci* **59:** (8) 1241.

Klysik J. 2002. Mice and humans: Chromosome engineering and its applications to functional genomics (Review). *Acta Biochim Pol* **49:** (3) 553.

Ko YG, Park H, Kim S. 2002. Novel regulatory interactions and activities of mammalian tRNA synthetases: Review. *Proteomics* **2:** (9) 1304.

Kohlmann A, Schoch C, Schnittger S, Kern W, Haferlach T. 2002. Diagnosis of leukemia using microarray technology (Review) (German, English Abstract). *Dtsch Med Wochenschr* **127:** (42) 2216.

Koonin EV, Wolf YI, Karev GP. 2002. The structure of the protein universe and genome evolution. *Nature* **420:** (6912) 218.

Kuo WP, Whipple ME, Sonis ST, Ohno-Machado L, Jenssen TK. 2002. Gene expression profiling by DNA microarrays and its application to dental research (Review). *Oral Oncol* **38:** (7) 650.

Lal SP, Christopherson RI, Dos Remedios CG. 2002. Antibody arrays: An embryonic but rapidly growing technology (Review). *Drug Discov Today* **7:** (18) S143.

Liao VHC, Freedman JH. 2002. Differential display analysis of gene expression in invertebrates (Review). *Cell Mol Life Sci* **59:** (8) 1256.

Michener CM, Ardekani AM, Petricoin EF, Liotta LA, Kohn EC. 2002. Genomics and proteomics: Application of novel technology to early detection and prevention of cancer. *Cancer Detect Prev* **26:** (4) 249.

Mira A, Klasson L, Andersson SGE. 2002. Microbial genome evolution: Sources of variability. *Curr Opin Microbiol* **5:** (5) 506.

Mo WJ, Karger BL. 2002. Analytical aspects of mass spectrometry and proteomics. *Curr Opin Chem Biol* **6:** (5) 666.

Nicholson JK, Connelly J, Lindon JC, Holmes E. 2002. Metabonomics: A platform for studying during toxicity and gene function. *Nat Rev Drug Discov* **1:** (2) 153.

Petricoin EF, Zoon KC, Kohn EC, Barrett JC, Liotta LA. 2002. Clinical proteomics: Translating benchside promise into bedside reality (Review). *Nat Rev Drug Discov* **1:** (9) 683.

Roses AD. 2002. Genome-based pharmacogenetics and the pharmaceutical industry (Review). *Nat Rev Drug Discov* **1:** (7) 541.

Ruijter JM, Van Kampen AHC, Baas F. 2002. Statistical evaluation of SAGE libraries: consequences for experimental design (Review). *Physiol Genomics* **11:** (2) 37.

Schaeferling M, Schiller S, Paul H, Kruschina M, Pavlickova P, Meerkamp M, Giammasi C, Kambhampati D. 2002. Application of self-assembly techniques in the design of biocompatible protein microarray surfaces (Review). *Electrophoresis* **23:** (18) 3097.

Shen YF, Smith RD. 2002. Proteomics based on high-efficiency capillary separations (Review). *Electrophoresis* **23:** (18) 3106.

Simon HG. 2002. Messenger RNA differential display strategies in birds and amphibians (Review). *Cell Mol Life Sci* **59:** (8) 1264.

Sreenivasulu N, Kishor PBK, Varshney RK, Altschmied L. 2002. Mining functional information from cereal genomes: The utility of expressed sequence tags (Review). *Curr Sci* **83:** (8) 965.

Stein J, Liang P. 2002. Differential display technology: A general guide (Review). *Cell Mol Life Sci* **59:** (8) 1235.

In order to keep subscribers up-to-date with the latest developments in their field, this current awareness service is provided by John Wiley & Sons and contains newly-published material on comparative and functional genomics. Each bibliography is divided into 16 sections. 1 Reviews & symposia; 2 General; 3 Large-scale sequencing and mapping; 4 Evolutionary genomics; 5 Comparative genomics; 6 Pathways, gene families and regulons; 7 Pharmacogenomics; 8 EST, cDNA and other clone resources; 9 Functional genomics; 10 Transcriptomics; 11 Proteomics; 12 Protein structural genomics; 13 Metabolomics; 14 Genomic approaches to development; 15 Technological advances; 16 Bioinformatics. Within each section, articles are listed in alphabetical order with respect to author. If, in the preceding period, no publications are located relevant to any one of these headings, that section will be omitted.

Copyright © 2003 John Wiley & Sons, Ltd.

Stein S, Liang P. 2002. Differential display analysis of gene expression in mammals: A *p53* story (Review). *Cell Mol Life Sci* **59:** (8) 1274.
Tsai YJ, Hoyme HE. 2002. Pharmacogenomics: The future of drug therapy (Mini-Review). *Clin Genet* **62:** (4) 257.
Tucker CL. 2002. High-throughput cell-based assays in yeast (Review). *Drug Discov Today* **7:** (18) S125.
Tunlid A, Talbot NJ. 2002. Genomics of parasitic and symbiotic fungi. *Curr Opin Microbiol* **5:** (5) 513.
Urnov FD, Rebar EJ. 2002. Designed transcription factors as tools for therapeutics and functional genomics. *Biochem Pharmacol* **64:** (5-6) 919.
VanSluys MA, Monteiro-Vitorello CB, Camargo LEA, Menck CFM, Da Silva ACR, Ferro JA, Oliveira MC, Setubal JC, Kitajima JP, Simpson AJ. 2002. Comparative genomic analysis of plant-associated bacteria. *Annu Rev Phytopathol* **40:** 169.
Vivarest CP, Gouy M, Thomarat F, Metenier G. 2002. Functional and evolutionary analysis of a eukaryotic parasitic genome. *Curr Opin Microbiol* **5:** (5) 499.
Wu W, Hu W, Kavanagh JJ. 2002. Proteomics in cancer research (Review). *Int J Gynecol Cancer* **12:** (5) 409.
Yamazaki M, Saito K. 2002. Differential display analysis of gene expression in plants (Review). *Cell Mol Life Sci* **59:** (8) 1246.
Yano H, Kuroda S, Buchanan BB. 2002. Disulfide proteome in the analysis of protein function and structure: Review. *Proteomics* **2:** (9) 1090.
Yao T. 2002. Bioinformatics for the genomic sciences and towards systems biology. Japanese activities in the post-genome era (Review). *Prog Biophys Mol Biol* **80:** (1-2) 23.
Ye SQ, Usher DC, Zhang LQ. 2002. Gene expression profiling of human diseases by serial analysis of gene expression (Review). *J Biomed Sci* **9:** (5) 384.

### 3 Large-scale sequencing and mapping

Ajdic D, McShan WM, McLaughlin RE, Savic G, Chang J, Carson MB, Primeaux C, Tian RY, Kenton S, Jia HG *et al*. 2002. Genome sequence of *Streptococcus mutans* UA159, a cariogenic dental pathogen. *Proc Natl Acad Sci U S A* **99:** (22) 14434.
El Osta YGA, Hillier AJ, Davidson BE, Dobos M. 2002. Pulsed-field gel electrophoretic analysis of the genome of *Lactobacillus gasseri* ATCC33323 and construction of a physical map. *Electrophoresis* **23:** (19) 3321.
Feng Q, Zhang YJ, Hao P, Wang SY, Fu G, Huang YC, Li Y, Zhu JJ, Liu YL, Hu X *et al*. 2002. Sequence and analysis of rice chromosome 4. *Nature* **420:** (6913) 316.
Glaser P, Rusniok C, Buchrieser C, Chevalier F, Frangeul L, Msadek T, Zouine M, Couve E, Lalioui L, Poyart C *et al*. 2002. Genome sequence of *Streptococcus agalactiae*, a pathogen causing invasive neonatal disease. *Mol Microbiol* **45:** (6) 1499.
Nakamura Y, Kaneko T, Sato S, Ikeuchi M, Katoh H, Sasamoto S, Watanabe A, Iriguchi M, Kawashima K, Kimura T *et al*. 2002. Complete genome structure of the thermophilic cyanobacterium *Thermosynechococcus elongatus* BP-1. *DNA Res* **9:** (4) 123.
Raymond J, Zhaxybayeva O, Gogarten JP, Gerdes SY, Blankenship RE. 2002. Whole-genome analysis of photosynthetic prokaryotes. *Science* **298:** (5598) 1616.
Sasaki T, Matsumoto T, Yamamoto K, Sakata K, Baba T, Katayose Y, Wu JZ, Niimura Y, Cheng ZK, Nagamura Y *et al*. 2002. The genome sequence and structure of rice chromosome 1. *Nature* **420:** (6913) 312.
Schell MA, Karmirantzou M, Snel B, Vilanova D, Berger B, Pessi G, Zwahlen MC, Desiere F, Bork P, Delley M *et al*. 2002. The genome sequence of *Bifidobacterium longum* reflects its adaptation to the human gastrointestinal tract. *Proc Natl Acad Sci U S A* **99:** (22) 14422.
Zhang ZL, Harrison P, Gerstein M. 2002. Identification and analysis of over 2000 ribosomal protein pseudogenes in the human genome. *Genome Res* **12:** (10) 1466.

### 4 Evolutionary genomics

Ling LJ, Wang JH, Cui Y, Li W, Chen RS. 2002. Proteome-wide analysis of protein function composition reveals the clustering and phylogenetic properties of organisms. *Mol Phylogenet Evol* **25:** (1) 101.
Martin W, Rujan T, Richly E, Hansen A, Cornelsen S, Lins T, Leister D, Stoebe B, Hasegawa M, Penny D. 2002. Evolutionary analysis of Arabidopsis, cyanobacterial, and chloroplast genomes reveals plastid phylogeny and thousands of cyanobacterial genes in the nucleus. *Proc Natl Acad Sci U S A* **99:** (19) 12246.
Zo YG, Rivera ING, Russek-Cohen E, Islam MS, Siddique AK, Yunus M, Sack RB, Huq A, Colwell RR. 2002. Genomic profiles of clinical and environmental isolates of *Vibrio cholerae* O1 in choleraendemic areas of Bangladesh. *Proc Natl Acad Sci U S A* **99:** (19) 12409.

### 5 Comparative genomics

Bhattacharyya A, Stilwagen S, Reznik G, Feil H, Feil WS, Anderson I, Bernal A, D'Souza M, Ivanova N, Kapatral V *et al*. 2002. Draft sequencing and comparative genomics of *Xylella fastidiosa* strains reveal novel biological insights. *Genome Res* **12:** (10) 1556.
Cole ST. 2002. Comparative and functional genomics of the *Mycobacterium tuberculosis* complex. *Microbiology* **148:** (10) 2919.
Desiere F, Lucchini S, Canchaya C, Ventura M, Brussow H. 2002. Comparative genomics of phages and prophages in lactic acid bacteria. *Antonie van Leeuwenhoek* **82:** (1-4) 73.
Dullaghan EM, Malloff CA, Li AH, Lam WL, Stokes RW. 2002. Two-dimensional bacterial genome display: A method for the genomic analysis of mycobacteria. *Microbiology* **148:** (10) 3111.
Jin Q, Yuan ZH, Xu JG, Wang Y, Shen Y, Lu WC, Wang JH, Liu H, Yang J, Yang F et al. 2002. Genome sequence of *Shigella flexneri* 2a: Insights into pathogenicity through comparison with genomes of *Escherichia coli* K12 and O157. *Nucleic Acids Res* **30:** (20) 4432.
Liu SV, Saunders NJ, Jeffries A, Rest RF. 2002. Genome analysis and strain comparison of Correia repeats and Correia repeat-enclosed elements in pathogenic *Neisseria*. *J Bacteriol* **184:** (22) 6163.
Paulsen IT, Seshadri R, Nelson KE, Eisen JA, Heidelberg JF, Read TD, Dodson RJ, Umayam L, Brinkac LM, Beanan MJ et al. 2002. The Brucella suis genome reveals fundamental similarities between animal and plant pathogens and symbionts. *Proc Natl Acad Sci U S A* **99:** (20) 13148.
Sankar N, Machado J, Abdulla P, Hilliker AJ, Coe IR. 2002. Comparative genomic analysis of equilibrative nucleoside transporters suggests conserved protein structure despite limited sequence identity. *Nucleic Acids Res* **30:** (20) 4339.
Tettelin H, Masignani V, Cieslewicz MJ, Eisen JA, Peterson S, Wessels MR, Paulsen IT, Nelson KE, Margarit I, Read TD *et al*. 2002. Complete genome sequence and comparative genomic analysis of an emerging human pathogen, serotype V *Streptococcus agalactiae*. *Proc Natl Acad Sci U S A* **99:** (19) 12391.
Tsolis RM. 2002. Comparative genome analysis of the α-proteobacteria: Relationships between plant and animal host specificity. *Proc Natl Acad Sci U S A* **99:** (20) 12503.
Vandepoele K, Saeys Y, Simillion C, Raes J, Van de Peer Y. 2002. The automatic detection of homologous regions (ADHoRe) and its application to microcolinearity between *Arabidopsis* and rice. *Genome Res* **12:** (11) 1792.
Werner-Washburne M, Wylie B, Boyack K, Fuge E, Galbraith J, Weber J, Davidson G. 2002. Comparative analysis of multiple genome-scale data sets. *Genome Res* **12:** (10) 1564.

### 6 Pathways, gene families and regulons

Chou KC, Elrod DW. 2002. Bioinformatical analysis of G-protein-coupled receptors. *J Proteome Res* **1:** (5) 429.
Hekmat-Scafe DS, Scafe CR, McKinney AJ, Tanouye MA. 2002. Genome-wide analysis of the odorant-binding protein gene family in *Drosophila melanogaster*. *Genome Res* **12:** (9) 1357.
Kallberg Y, Oppermann U, Jornvall H, Persson B. 2002. Short-chain dehydrogenases/reductases (SDRs): Coenzyme-based functional assignments in completed genomes. *Eur J Biochem* **269:** (18) 4409.
Larsen CN, Wang HL. 2002. The ubiquitin superfamily: Members, features, and phylogenies. *J Proteome Res* **1:** (5) 411.
Wilson JW, Ramamurthy R, Porwollik S, McClelland M, Hammond T, Allen P, Ott CM, Pierson DL, Nickerson CA. 2002. Microarray analysis identifies *Salmonella* genes belonging to the low-shear modeled microgravity regulon. *Proc Natl Acad Sci U S A* **99:** (21) 13807.

Copyright © 2003 John Wiley & Sons, Ltd. 

### 7 Pharmacogenomics

Azuaje F. 2002. *In silico* approaches to micro array-based disease classification and gene function discovery. *Ann Med* **34:** (4) 299.

Baron A, Scarpa A. 2002. Gene expression profiling as a tool for the identification of molecular targets. *Tumori* (Suppl) S17.

Calvo A, Xiao NQ, Jang J, Best CJM, Leiva I, Emmert-Buck MR, Jorcyk C, Green JE. 2002. Alterations in gene expression profiles during prostate cancer progression: Functional correlations to tumorigenicity and down-regulation of selenoprotein-P in mouse and human tumors. *Cancer Res* **62:** (18) 5325.

Charlwood J, Skehel JM, King N, Camilleri P, Lord P, Bugelski P, Atif U. 2002. Proteomic analysis of rat kidney cortex following treatment with gentamicin. *J Proteome Res* **1:** (1) 73.

Colangelo V, Schurr J, Ball MJ, Pelaez RP, Bazan NG, Lukiw WJ. 2002. Gene expression profiling of 12633 genes in Alzheimer hippocampal CA1: Transcription and neurotrophic factor down-regulation and up-regulation of apoptotic and pro-inflammatory signaling. *J Neurosci Res* **70:** (3) 462.

Crabb JW, Miyagi M, Gu XR, Shadrach K, West KA, Sakaguchi H, Kamei M, Hasan A, Yan L, Rayborn ME *et al*. 2002. Drusen proteome analysis: An approach to the etiology of age-related macular degeneration. *Proc Natl Acad Sci U S A* **99:** (23) 14682.

Dare TO, Davies HA, Turton JA, Lomas L, Williams TC, York MJ. 2002. Application of surface-enhanced laser desorption/ionization technology to the detection and identification of urinary parvalbumin-α: A biomarker of compound-induced skeletal muscle toxicity in the rat. *Electrophoresis* **23:** (18) 3241.

Dayal B, Ertel NH. 2002. ProteinChip technology: A new and facile method for the identification and measurement of high-density lipoproteins apoA-I and apoA-II and their glycosylated products in patients with diabetes and cardiovascular disease. *J Proteome Res* **1:** (4) 375.

De Vos J, Thykjaer T, Tarte K, Ensslen M, Raynaud P, Requirand G, Pellet F, Pantesco, Reme T, Jourdan M *et al*. 2002. Comparison of gene expression profiling between malignant and normal plasma cells with oligonucleotide arrays. *Oncogene* **21:** (44) 6848.

Diehl S, Diehl F, El Sayed NM, Clayton C, Hoheisel JD. 2002. Analysis of stage-specific gene expression in the bloodstream and the procyclic form of *Trypanosoma brucei* using a genetic DNA-microarray. *Mol Biochem Parasitol* **123:** (2) 115.

Eikmans M, Baelde HJ, De Heer E, Bruijn JA. 2002. RNA expression profiling as prognostic tool in renal patients: Toward nephrogenomics. *Kidney Int* **62:** (4) 1125.

Francioso F, Carinci F, Tosi L, Scapoli L, Pezzetti F, Passerella E, Evangelisti R, Pastore A, Pelucchi S, Piattelli A *et al*. 2002. Identification of differentially expressed genes in human salivary gland tumors by DNA microarrays. *Mol Cancer Ther* **1:** (7) 533.

Gaubin Y, Manceau C, Skandrani D, Croute F, Murat JC, Soleilhavoup JP. 2002. Changes in level of genes expression after exposure of cultured human cells to endosulfan as investigated by a cDNA microarrays method. *Fresenius Environ Bull* **11:** (8) 466.

Hathout Y, Riordan K, Gehrmann M, Fenselau C. 2002. Differential protein expression in the cytosol fraction of an MCF-7 breast cancer cell line selected for resistance toward melphalan. *J Proteome Res* **1:** (5) 435.

Hiltunen MO, Tuomisto TT, Niemi M, Brasen JH, Rissanen TT, Toronen P, Vajanto I, Yla-Herttuala S. 2002. Changes in gene expression in atherosclerotic plaques analyzed using DNA array. *Atherosclerosis* **165:** (1) 23.

Hu S, Jiang J, Cook LM, Richards DP, Horlick L, Wong B, Dovichi NJ. 2002. Capillary sodium dodecyl sulfate-DALT electrophoresis with laser-induced fluorescence detection for size-based analysis of proteins in human colon cancer cells. *Electrophoresis* **23:** (18) 3136.

Inoue A, Yoshida N, Omoto Y, Oguchi S, Yamori T, Kiyama R, Hayashi S. 2002. Development of cDNA microarray for expression profiling of estrogen-responsive genes. *J Mol Endocrinol* **29:** (2) 175.

Karjalainen HM, Sironen RK, Elo MA, Kaarniranta K, Takigawa M, Helminen HJ, Lammi MJ. 2002. Gene expression profiles in chondrosarcoma cells subjected to cyclic stretching and hydrostatic pressure. A cDNA array study. *Biorheology* **40:** (1-3) 93.

Kirkwood SC, Hockett RD. 2002. Pharmacogenomic biomarkers. *Dis Marker* **18:** (2) 63.

Korenberg MJ. 2002. Prediction of treatment response using gene expression profiles. *J Proteome Res* **1:** (1) 55.

Lankford AR, Byford AM, Ashton KJ, French BA, Lee JK, Headrick JP, Matherne GP. 2002. Gene expression profile of mouse myocardium with transgenic overexpression of $A_1$ adenosine receptors. *Physiol Genomics* **11:** (2) 81.

Lee S, Baek M, Yang H, Bang YJ, Kim WH, Ha JH, Kim DK, Jeoung DI. 2002. Identification of genes differentially expressed between gastric cancers and normal gastric mucosa with cDNA microarrays. *Cancer Lett* **184:** (2) 197.

Li X, Gu W, Masinde G, Covarrubias M, Mohan S, Baylink DJ. 2002. Temporal analysis of gene expression by microarray during wound healing. *Wounds Compend Clin Res Pract* **14:** (2) 67.

Lim JWE, Bodnar A. 2002. Proteome analysis of conditioned medium from mouse embryonic fibroblast feeder layers which support the growth of human embryonic stem cells. *Proteomics* **2:** (9) 1187.

Lin JZ, Tsuboi Y, Pan W, Giebink GS, Adams GL, Kim Y. 2002. Analysis by cDNA microarrays of altered gene expression in middle ears of rats following pneumococcal infection. *Int J Pediatr Otorhinolaryngol* **65:** (3) 203.

McCormick SM, Frye SR, Eskin SG, Teng CL, Lu CM, Russell CG, Chittur KK, McIntire LV. 2002. Microarray analysis of shear stressed endothelial cells. *Biorheology* **40:** (1-3) 5.

McDonald WH, Yates JR. 2002. Shotgun proteomics and biomarker discovery. *Dis Marker* **18:** (2) 99.

Morgan KT, Ni H, Brown HR, Yoon L, Qualls CW, Crosby LM, Reynolds R, Gaskill B, Anderson SP, Kepler TB *et al*. 2002. Application of cDNA microarray technology to *in vitro* toxicology and the selection of genes for a real-time RT-PCR-based screen for oxidative stress in Hep-G2 cells. *Toxicol Pathol* **30:** (4) 435.

Nagayama S, Katagiri T, Tsunoda T, Hosaka T, Nakashima Y, Araki N, Kusuzaki K, Nakayama T, Tsuboyama T, Nakamura T *et al*. 2002. Genome-wide analysis of gene expression in synovial sarcomas using a cDNA microarray. *Cancer Res* **62:** (20) 5859.

Okutsu J, Tsunoda T, Kaneta Y, Katagiri T, Kitahara O, Zembutsu H, Yanagawa R, Miyawaki S, Kuriyama K, Kubota N *et al*. 2002. Prediction of chemosensitivity for patients with acute myeloid leukemia, according to expression levels of 28 genes selected by genome-wide complementary DNA microarray analysis. *Mol Cancer Ther* **1:** (12) 1035.

Pavlickova P, Lensen NM, Paul H, Schaeferling M, Giammasi C, Kruschina M, Du WD, Theisen M, Ibba M, Ortigao F *et al*. 2002. Antibody detection in human serum using a versatile protein chip platform constructed by applying nanoscale self-assembled architectures on gold. *J Proteome Res* **1:** (3) 227.

Petricoin EF, Ornstein DK, Paweletz CP, Ardekani A, Hackett PS, Hitt BA, Velassco A, Trucco C, Wiegand L, Wood K *et al*. 2002. Serum proteomic patterns for detection of prostate cancer. *J Nat Cancer Inst* **94:** (20) 1576.

Prasan AM, McCarron HCK, White MY, McLennan SV, Tchen AS, Hambly BD, Jeremy RW. 2002. Duration of ischaemia determines matrix metalloproteinase-2 activation in the reperfused rabbit heart. *Proteomics* **2:** (9) 1204.

Rogers PD, Barker KS. 2002. Evaluation of differential gene expression in fluconazole-susceptible and -resistant isolates of *Candida albicans* by cDNA microarray analysis. *Antimicrob Agents Chemother* **46:** (11) 3412.

Sarang SS, Yoshida T, Cadet R, Valeras AS, Jensen RV, Gullans SR. 2002. Discovery of molecular mechanisms of neuroprotection using cell-based bioassays and oligonucleotide arrays. *Physiol Genomics* **11:** (2) 45.

Sergeant GP, Large RJ, Beckett EAH, McGeough CM, Ward SM, Horowitz B. 2002. Microarray comparison of normal and *W/W$^{v}$* mice in the gastric fundus indicates a supersensitive phenotype. *Physiol Genomics* **11:** (1) 1.

Shan L, He M, Yu MS, Qiu CP, Lee NH, Liu ET, Snyderwine EG. 2002. cDNA microarray profiling of rat mammary gland carcinomas induced by 2-amino-1 methyl-6-phenylimidazo[4,5-*b*]pyridine and 7, 12-dimethylbenz[*a*]anthracene. *Carcinogenesis* **23:** (10) 1561.

Sinz A, Bantscheff M, Mikkat S, Ringel B, Drynda S, Kekow J, Thiesen HJ, Glocker MO. 2002. Mass spectrometric proteome analyses of synovial fluids and plasmas from patients suffering from rheumatoid arthritis and comparison to reactive arthritis or osteoarthritis. *Electrophoresis* **23:** (19) 3445.

Copyright © 2003 John Wiley & Sons, Ltd. 

Struchkov VA, Strazhevskaya NB, Zhdanov RI. 2002. DNA-bound lipids of normal and tumor cells: Retrospective and outlooks for functional genomics. *Bioelectrochemistry* **58:** (1) 23.

Sun DX, Lennernas H, Welage LS, Barnett JL, Landowski CP, Foster D, Fleisher D, Lee KD, Amidon GL. 2002. Comparison of human duodenum and Caco-2 gene expression profiles for 12,000 gene sequences tags and correlation with permeability of 26 drugs. *Pharmaceut Res* **19:** (10) 1400.

Sun YH, Yang Q, Wang LH, Gao L, Tang R, Ying K, Xu CL, Qian SX, Li Y, Xie Y *et al*. 2002. Monitoring gene expression profile changes in bladder transitional cell carcinoma using cDNA microarray. *Urol Oncol* **7:** (5) 207.

Tani TH, Khodursky A, Blumenthal RM, Brown PO, Matthews RG. 2002. Adaptation to famine: A family of stationary-phase genes revealed by microarray analysis. *Proc Natl Acad Sci U S A* **99:** (21) 13471.

Thongboonkerd V, Gozal E, Sachleben LR, Arthur JM, Pierce WM, Cai J, Chao J, Bader M, Pesquero JB, Gozal D *et al*. 2002. Proteomic analysis reveals alterations in the renal kallikrein pathway during hypoxia-induced hypertension. *J Biol Chem* **277:** (38) 34708.

Thorns C, Gaiser T, Lange K, Merz H, Feller AC. 2002. cDNA arrays: Gene expression profiles of Hodgkin's disease and anaplastic large cell lymphoma cell lines. *Pathol Int* **52:** (9) 578.

Tomlinson AJ, Hincapie M, Morris GE, Chicz RM. 2002. Global proteome analysis of a human gastric carcinoma. *Electrophoresis* **23:** (18) 3233.

Vawter MP, Crook JM, Hyde TM, Kleinman JE, Weinberger DR, Becker KG, Freed WJ. 2002. Microarray analysis of gene expression in the prefrontal cortex in schizophrenia: A preliminary study. *Schizophr Res* **58:** (1) 11.

Wada Y, Kayo T, Koizumi A. 2002. Characterization of gene expression profile associated with energy restriction-induced cold tolerance of heart. *Microsc Res Tech* **59:** (4) 313.

Wallqvist A, Rabow AA, Shoemaker RH, Sausville EA, Covell DG. 2002. Establishing connections between microarray expression data and chemotherapeutic cancer pharmacology. *Mol Cancer Ther* **1:** (5) 311.

Weinschenk T, Gouttefangeas U, Schirle M, Obermayr F, Walter S, Schoor O, Kurek R, Loeser W, Bichler KH, Wernet D *et al*. 2002. Integrated functional genomics approach for the design of patient-individual antitumor vaccines. *Cancer Res* **62:** (20) 5818.

Woodbury RL, Varnum SM, Zangar RC. 2002. Elevated HGF levels in sera from breast cancer patients detected using a protein microarray ELISA. *J Proteome Res* **1:** (3) 233.

Wu SL, Amato H, Biringer R, Choudhary G, Shieh P, Hancock WS. 2002. Targeted proteomics of low-level proteins in human plasma by LC/MS$^n$: Using human growth hormone as a model system. *J Proteome Res* **1:** (5) 459.

Yokoi A, Kuromitsu J, Kawai T, Nagasu T, Sugi NH, Yoshimatsu K, Yoshino H, Owa T. 2002. Profiling novel sulfonamide antitumor agents with cell-based phenotypic screens and array-based gene expression analysis. *Mol Cancer Ther* **1:** (4) 275.

Zeytun A, McKallip RJ, Fisher M, Camacho I, Nagarkatti M, Nagarkatti PS. 2002. Analysis of 2,3,7,8-tetrachlorodibenzo-*p*-dioxin-induced gene expression profile *in vivo* using pathway-specific cDNA arrays. *Toxicology* **178:** (3) 241.

Zheng X, Ravatn R, Lin Y, Shih WC, Rabson A, Strair R, Huberman E, Conney A, Chin KV. 2002. Gene expression of TPA induced differentiation in HL-60 cells by DNA microarray analysis. *Nucleic Acids Res* **30:** (20) 4489.

Zogakis TG, Costouros NG, Kruger EA, Forbes S, He M, Qian M, Feldman AL, Figg WD, Alexander HR, Liu ET *et al*. 2002. Microarray gene expression profiling of angiogenesis inhibitors using the rat aortic ring assay. *Biotechniques* **33:** (3) 664.

### 8 EST, cDNA and other clone resources

Asano T, Masumura T, Kusano H, Kikuchi S, Kurita A, Shimada H, Kadowaki K. 2002. Construction of a specialized cDNA library from plant cells isolated by laser capture microdissection: Toward comprehensive analysis of the genes expressed in the rice phloem. *Plant J* **32:** (3) 401.

Ayoubi P, Jin XJ, Leite S, Liu XH, Martajaja J, Abduraham A, Wan QL, Yan W, Misawa E, Prade RA. 2002. PipeOnline 2,0: automated EST processing and functional data sorting. *Nucleic Acids Res* **30:** (21) 4761.

Chen JJ, Sun M, Lee SG, Zhou GL, Rowley JD, Wang S. 2002. Identifying novel transcripts and novel genes in the human genome by using novel SAGE tags. *Proc Natl Acad Sci U S A* **99:** (19) 12257.

Ding KY, Sun XJ, Zhou M, Cai JJ, Cai TJ, Zhang Y, Zhu T, Zhang ZG, Qiang BQ, Shen Y. 2002. Identification of novel protein-coding genes from human fetal hippocampus ESTs database. *Neurosci Res Commun* **31:** (2) 93.

Dunn JJ, McCorkle SR, Praissman LA, Hind G, Van der Lelie D, Bahou WF, Gnatenko DV, Krause MK. 2002. Genomic signature tags (GSTs): A system for profiling genomic DNA. *Genome Res* **12:** (11) 1756.

Marvanova A, Toronen P, Storvik M, Lakso M, Castren E, Wong G. 2002. Synexpression analysis of ESTs in the rat brain reveals distinct patterns and potential drug targets. *Mol Brain Res* **104:** (2) 176.

Michiels F, Van Es H, Van Rompaey L, Merchiers P, Francken B, Pittois K, Van der Schueren J, Brys R, Vandersmissen J, Beirinckx F *et al*. 2002. Arrayed adenoviral expression libraries for functional screening. *Nat Biotechnol* **20:** (11) 1154.

Nakayama M, Kikuno R, Ohara O. 2002. Protein-protein interactions between large proteins: Two-hybrid screening using a functionally classified library composed of long cDNAs. *Genome Res* **12:** (11) 1773.

Ortega D, Raynal M, Laudie M, Llauro C, Cooke R, Devic M, Genestier S, Picard G, Abad P, Contard P *et al*. 2002. Flanking sequence tags in *Arabidopsis thaliana* T-DNA insertion lines: A pilot study. *C R Biol* **325:** (7) 773.

Szabados L, Kovacs I, Oberschall A, Abraham E, Kerekes I, Zsigmond L, Nagy R, Alvarado M, Krasovskaja I, Gal M *et al*. 2002. Distribution of 1000 sequenced T-DNA tags in the *Arabidopsis* genome. *Plant J* **32:** (2) 233.

Zeng S, Gong ZY. 2002. Expressed sequence tag analysis of expression profiles of zebrafish testis and ovary. *Gene* **294:** (1-2) 45.

### 9 Functional genomics

Backhus LE, DeRisi J, Brown PO, Bisson LF. 2001. Functional genomic analysis of a commercial wine strain of *Saccharomyces cerevisiae* under differing nitrogen conditions. *FEMS Yeast Res* **1:** (2) 111.

Bernaudin M, Tang Y, Reilly M, Petit E, Sharp FR. 2002. Brain genomic response following hypoxia and re-oxygenation in the neonatal rat: Identification of genes that might contribute to hypoxia-induced ischemic tolerance. *J Biol Chem* **277:** (42) 39728.

Forst CV. 2002. Network genomics: A novel approach for the analysis of biological systems in the post-genomic era. *Mol Biol Rep* **29:** (3) 265.

Jarvik JW, Fisher GW, Shi C, Hennen L, Hauser C, Adler S, Berget PB. 2002. *In vivo* functional proteomics: Mammalian genome annotation using CD-tagging. *Biotechniques* **33:** (4) 852.

Moore S, Vrebalov J, Payton P, Giovannoni J. 2002. Use of genomics tools to isolate key ripening genes and analyse fruit maturation in tomato. *J Exp Bot* **53:** (377) 2023.

Nielsen J, Olsson L. 2002. An expanded role for microbial physiology in metabolic engineering and functional genomics: Moving towards systems biology. *FEMS Yeast Res* **2:** (2) 175.

Pratt RJ, Aramayo R. 2002. Improving the efficiency of gene replacements in *Neurospora crassa*: A first step towards a large-scale functional genomics project. *Fungal Genet Biol* **37:** (1) 56.

Sasaki T. 2002. Rice genomics to understand rice plant as an assembly of genetic codes. *Curr Sci* **83:** (7) 834.

Xiao B, Wu ZG, Yang XS, Li GL, Xie GJ. 2002. Analysis of gene expression in genetic epilepsy-prone rat using a cDNA expression array. *Seizure Eur J Epilep* **11:** (7) 418.

Zhang MQ. 2002. Extracting functional information from microarrays: A challenge for functional genomics. *Proc Natl Acad Sci U S A* **99:** (20) 12509.

### 10 Transcriptomics

Aharoni A, O'Connell AP. 2002. Gene expression analysis of strawberry achene and receptacle maturation using DNA microarrays. *J Exp Bot*

Copyright © 2003 John Wiley & Sons, Ltd. 

**53:** (377) 2073.
Beckering CL, Steil L, Weber MHW, Volker U, Marahiel MA. 2002. Genomewide transcriptional analysis of the cold shock response in *Bacillus subtilis*. *J Bacteriol* **184:** (22) 6395.
Boeuf S, Keijer J, Franssen-Van Hal NLW, Klaus S. 2002. Individual variation of adipose gene expression and identification of covariated genes by cDNA microarrays. *Physiol Genomics* **11:** (1) 31.
Breyne P, Dreesen R, Vandepoele K, De Veylder L, Van Breusegem F, Callewaert L, Rombauts S, Raes J, Cannoot B, Engler G *et al*. 2002. Transcriptome analysis during cell division in plants. *Proc Natl Acad Sci U S A* **99:** (23) 14825.
Davoren JD, Nykiforuk CL, Laroche A, Weselake RJ. 2002. Sucrose-induced changes in the transcriptome of cell suspension cultures of oilseed rape reveal genes associated with lipid biosynthesis. *Plant Physiol Biochem* **40:** (9) 719.
De Vries RP, Jansen J, Aguilar G, Parenicova L, Joosten V, Wulfert F, Benen JAE, Visser J. 2002. Expression profiling of pectinolytic genes from *Aspergillus niger*. *FEBS Lett* **530:** (1-3) 41.
Fedorova M, Van de Mortel J, Matsumoto PA, Cho J, Town CD, Van den Bosch KA, Gantt JS, Vance CP. 2002. Genome-wide identification of nodule-specific transcripts in the model legume *Medicago truncatula*. *Plant Physiol* **130:** (2) 519.
Herrero J, Dopazo J. 2002. Combining hierarchical clustering and self-organizing maps for exploratory analysis of gene expression patterns. *J Proteome Res* **1:** (5) 467.
Katsanis N, Worley KC, Gonzalez G, Ansley SJ, Lupski JR. 2002. A computational/functional genomics approach for the enrichment of the retinal transcriptome and the identification of positional candidate retinopathy genes. *Proc Natl Acad Sci U S A* **99:** (22) 14326.
Klok EJ, Wilson IW, Wilson D, Chapman SC, Ewing RM, Somerville SC, Peacock WJ, Dolferus R, Dennis ES. 2002. Expression profile analysis of the low-oxygen response in *Arabidopsis* root cultures. *Plant Cell* **14:** (10) 2481.
Krasnova IN, Mccoy MT, Ladenheim B, Cadet JL. 2002. cDNA array analysis of gene expression profiles in the striata of wild-type and Cu/Zn superoxide dismutase transgenic mice treated with neurotoxic doses of amphetamine. *FASEB J* **16:** (11) 1379.
Kuipers OP, De Jong A, Baerends RJS, Van Hijum SAFT, Zomer AL, Karsens HA, Den Hengst CD, Kramer NE, Buist G, Kok J. 2002. Transcriptome analysis and related databases of *Lactococcus lactis*. *Antonie van Leeuwenhoek* **82:** (1-4) 113.
Lee CK, Allison DB, Brand J, Weindruch R, Prolla TA. 2002. Transcriptional profiles associated with aging and middle age-onset caloric restriction in mouse hearts. *Proc Natl Acad Sci U S A* **99:** (23) 14988.
Le Roch KG, Zhou YY, Batalov S, Winzeler EA. 2002. Monitoring the chromosome 2 intraerythrocytic transcriptome of *Plasmodium falciparum* using oligonucleotide arrays. *Am J Trop Med Hyg* **67:** (3) 233.
Lund J, Tedesco P, Duke K, Wang J, Kim SK, Johnson TE. 2002. Transcriptional profile of aging in *C. elegans*. *Curr Biol* **12:** (18) 1566.
Menges M, Hennig L, Gruissem W, Murray JAH. 2002. Cell cycle-regulated gene expression in *Arabidopsis*. *J Biol Chem* **277:** (44) 41987.
Moseyko N, Zhu T, Chang HS, Wang X, Feldman LJ. 2002. Transcription profiling of the early gravitropic response in *Arabidopsis* using high-density oligonucleotide probe microarrays. *Plant Physiol* **130:** (2) 720.
Nantel A, Dignard D, Bachewich C, Harcus D, Marcil A, Bouin AP, Sensen CW, Hogues H, Van het Hoog M, Gordon P, Rigby T, Benoit F, Tessier DC, Thomas DY, Whiteway M. 2002. Transcription profiling of *Candida albicans* cells undergoing the yeast-to-hyphal transition. *Mol Biol Cell* **13:** (10) 3452.
Oshima T, Aiba H, Masuda Y, Kanaya S, Sugiura M, Wanner BL, Mori H, Mizuno T. 2002. Transcriptome analysis of all two-component regulatory system mutants of *Escherichia coli* K12. *Mol Microbiol* **46:** (1) 281.
Piper MDW, Daran-Lapujade P, Bro C, Regenberg B, Knudsen S, Nielsen J, Pronk JT. 2002. Reproducibility of oligonucleotide microarray transcriptome analyses: An interlaboratory comparison using chemostat cultures of *Saccharomyces cerevisiae*. *J Biol Chem* **277:** (40) 37001.
Talaat AM, Howard ST, Halel W, Lyons R, Garner H, Johnston SA. 2002. Genomic DNA standards for gene expression profiling in *Mycobacterium tuberculosis*: Online art. no. e104. *Nucleic Acids Res* **30:** (20) e104.
Wicker N, Dembele D, Raffelsberger W, Poch O. 2002. Density of points clustering, application to transcriptomic data analysis. *Nucleic Acids Res* **30:** (18) 3992.
Zhang XS, Chen ZA, Huang H, Gordon JR, Xiang J. 2002. DNA microarray analysis of the gene expression profiles of naive *versus* activated tumor-specific T-cells. *Life Sci* **71:** (25) 3005.

### 11 Proteomics

Andon NL, Hollingworth S, Koller A, Greenland AJ, Yates JR, Haynes PA. 2002. Proteomic characterization of wheat amyloplasts using identification of proteins by tandem mass spectrometry. *Proteomics* **2:** (9) 1156.
Arthur JM, Thongboonkerd V, Scherzer JA, Cai J, Pierce WM, Klein JB. 2002. Differential expression of proteins in renal cortex and medulla: A proteomic approach. *Kidney Int* **62:** (4) 1314.
Baty JW, Hampton MB, Winterbourn CC. 2002. Detection of oxidant sensitive thiol proteins by fluorescence labeling and two-dimensional electrophoresis. *Proteomics* **2:** (9) 1261.
Borodovsky A, Ovaa H, Kolli N, Gan-Erdene T, Wilkinson KD, Ploegh HL, Kessler BM. 2002. Chemistry-based functional proteomics reveals novel members of the deubiquitinating enzyme. *Chem Biol* **9:** (10) 1149.
Bradley BP, Shrader EA, Kimmel DG, Meiller JC. 2002. Protein expression signatures: An application of proteomics. *Mar Environ Res* **54:** (3-5) 373.
Chan LL, Lo SCL, Hodgkiss IJ. 2002. Proteomic study of a model causative agent of harmful red tide, *Prorocentrum triestinum* I: Optimization of sample preparation methodologies for analyzing with two-dimensional electrophoresis. *Proteomics* **2:** (9) 1169.
Chen W, Ji JG, Zhao R, Ru BG. 2002. Comparative proteome analysis of human temporal cortex lobes by two-dimensional electrophoresis and identification of selected common proteins. *Neurochem Res* **27:** (9) 871.
Chen ZY, Brown RL, Damann KE, Cleveland TE. 2002. Identification of unique or elevated levels of kernel proteins in aflatoxin-resistant maize genotypes through proteome analysis. *Phytopathology* **92:** (10) 1084.
Cheung PY, Lai WP, Lau HY, Lo SCL, Wong MS. 2002. Acute and chronic effect of dietary phosphorus restriction on protein expression in young rat renal proximal tubules. *Proteomics* **2:** (9) 1211.
Cordwell SJ, Larsen MR, Cole RT, Walsh BJ. 2002. Comparative proteomics of *Staphylococcus aureus* and the response of methicillin-resistant and methicillin-sensitive strains to Triton X-100. *Microbiology* **148:** (9) 2765.
Devreese B, Janssen KPC, Vanrobaeys F, Van Herp F, Martens GJM, Van Beeumen J. 2002. Automated nanoflow liquid chromatography-tandem mass spectrometry for a differential display proteomic study on *Xenopus laevis* neuroendocrine cells. *J Chromatogr A* **976:** (1-2) 113.
Ding YHR, Zhang SP, Tomb JF, Ferry JG. 2002. Genomic and proteomic analyses reveal multiple homologs of genes encoding enzymes of the methanol:coenzyme M methyltransferase system that are differentially expressed in methanol- and acetate-grown *Methanosarcina thermophila*. *FEMS Microbiol Lett* **215:** (1) 127.
Duche O, Tremoulet F, Namane A, Labadie J. 2002. A proteomic analysis of the salt stress response of *Listeria monocytogenes*. *FEMS Microbiol Lett* **215:** (2) 183.
Flanagan LM, Plowman JE, Bryson WG. 2002. The high sulphur proteins of wool: Towards an understanding of sheep breed diversity. *Proteomics* **2:** (9) 1240.
Gozal E, Gozal D, Pierce WM, Thongboonkerd V, Scherzer JA, Sachleben LR, Brittian KR, Guo SZ, Cai J, Klein JB. 2002. Proteomic analysis of CA1 and CA3 regions of rat hippocampus and differential susceptibility to intermittent hypoxia. *J Neurochem* **83:** (2) 331.
Hixson KK, Rodriguez N, Camp DG, Strittmatter EF, Lipton MS, Smith RD. 2002. Evaluation of enzymatic digestion and liquid chromatography-mass spectrometry peptide mapping of the integral membrane protein bacteriorhodopsin. *Electrophoresis* **23:** (18) 3224.
Huang YH, Chang AYW, Huang CM, Huang SW, Chan SHH. 2002. Proteomic analysis of lipopolysaccharide-induced apoptosis in PC12 cells. *Proteomics* **2:** (9) 1220.
Islam N, Woo SH, Tsujimoto H, Kawasaki H, Hirano H. 2002. Proteome approaches to characterize seed storage proteins related to ditelocentric

Copyright © 2003 John Wiley & Sons, Ltd. 

chromosomes in common wheat (*Triticum aestivum* L). *Proteomics* **2:** (9) 1146.
Jiang H, English AM. 2002. Quantitative analysis of the yeast proteome by incorporation of isotopically labeled leucine. *J Proteome Res* **1:** (4) 345.
Karty JA, Ireland MME, Brun YV, Reilly JP. 2002. Defining absolute confidence limits in the identification of *Caulobacter* proteins by peptide mass mapping. *J Proteome Res* **1:** (4) 325.
Korolainen MA, Goldsteins G, Alafuzoff I, Koistinaho J, Pirttila T. 2002. Proteomic analysis of protein oxidation in Alzheimer's disease brain. *Electrophoresis* **23:** (19) 3428.
Krogan NJ, Kim M, Ahn SH, Zhong GQ, Kobor MS, Cagney G, Emili A, Shilatifard A, Buratowski S, Greenblatt JF. 2002. RNA polymerase II elongation factors of *Saccharomyces cerevisiae*: A targeted proteomics approach. *Mol Cell Biol* **22:** (20) 6979.
Lind C, Gerdes R, Hamnell Y, Schuppe-Koistinen I, Von Lowenhielm HB, Holmgren A, Cotgreave IA. 2002. Identification of *S*-glutathionylated cellular proteins during oxidative stress and constitutive metabolism by affinity purification and proteomic analysis. *Arch Biochem Biophys* **406:** (2) 229.
Low TY, Seow TK, Chung MCM. 2002. Separation of human erythrocyte membrane associated proteins with one-dimensional and two-dimensional gel electrophoresis followed by identification with matrix-assisted laser desorption/ionization-time of flight mass spectrometry. *Proteomics* **2:** (9) 1229.
Lum JHK, Fung KL, Cheung PY, Wong MS, Lee CH, Kwok FSL, Leung MCP, Hui PK, Lo SCL. 2002. Proteome of oriental ginseng *Panax ginseng* C. A. Meyer and the potential to use it as an identification tool. *Proteomics* **2:** (9) 1123.
Melin P, Schnurer J, Wagner EGH. 2002. Proteome analysis of *Aspergillus nidulans* reveals proteins associated with the response to the antibiotic concanamycin A, produced by *Streptomyces* species. *Mol Genet Genomics* **267:** (6) 695.
Navas A, Lopez JA, Esparrago G, Camafeita E, Albar JP. 2002. Protein variability in *Meloidogyne* spp. (Nematoda: Meloidogynidae) revealed by two-dimensional gel electrophoresis and mass spectrometry. *J Proteome Res* **1:** (5) 421.
Nebl T, Pestonjamasp KN, Leszyk JD, Crowley JL, Oh SW, Luna EJ. 2002. Proteomic analysis of a detergent resistant membrane skeleton from neutrophil plasma membranes. *J Biol Chem* **277:** (45) 43399.
Nouwens AS, Willcox MDP, Walsh BJ, Cordwell SJ. 2002. Proteomic comparison of membrane and extracellular proteins from invasive (PAO1) and cytotoxic (6206) strains of *Pseudomonas aeruginosa*. *Proteomics* **2:** (9) 1325.
Pang JX, Ginanni N, Dongre AR, Hefta SA, Opiteck GJ. 2002. Biomarker discovery in urine by proteomics. *J Proteome Res* **1:** (2) 161.
Reynolds KJ, Yao XD, Fenselau C. 2002. Proteolytic $^{18}O$ labeling for comparative proteomics: Evaluation of endoprotease Glu-C as the catalytic agent. *J Proteome Res* **1:** (1) 27.
Salekdeh GH, Siopongco J, Wade LJ, Ghareyazie B, Bennett J. 2002. Proteomic analysis of rice leaves during drought stress and recovery. *Proteomics* **2:** (9) 1131.
Sato K, Iwasaki T, Sakakibara K, Itakura S, Fukami Y. 2002. Towards the molecular dissection of fertilization signaling: Our functional genomic/proteomic strategies. *Proteomics* **2:** (9) 1079.
Shen SH, Matsubae M, Takao T, Tanaka N, Komatsu S. 2002. A proteomic analysis of leaf sheaths from rice. *J Biochem* **132:** (4) 613.
Sinchaikul S, Sookkheo B, Phutrakul S, Pan FM, Chen ST. 2002. Proteomic study of cold shock protein in *Bacillus stearothermophilus* P1: Comparison of temperature downshifts. *Proteomics* **2:** (9) 1316.
Sookkheo B, Sinchaikul S, Thannan H, Thongprasong O, Phutrakul S, Chen ST. 2002. Proteomic analysis of a thermostable superoxide dismutase from *Bacillus stearothermophilus* TLS33. *Proteomics* **2:** (9) 1311.
Tan YP, Lin Q, Wang XH, Joshi S, Hew CL, Leung KY. 2002. Comparative proteomic analysis of extracellular proteins of *Edwardsiella tarda*. *Infec Immun* **70:** (11) 6475.
Taylor SW, Warnock DE, Glenn GM, Zhang B, Fahy E, Gaucher SP, Capaldi RA, Gibson BW, Ghosh SS. 2002. An alternative strategy to determine the mitochondrial proteome using sucrose gradient fractionation and 1D PAGE on highly purified human heart mitochondria. *J Proteome Res* **1:** (5) 451.
Thongboonkerd V, McIeish KR, Arthur JM, Klein JB. 2002. Proteomic analysis of normal human urinary proteins isolated by acetone precipitation or ultracentrifugation. *Kidney Int* **62:** (4) 1461.
Tremoulet F, Duche O, Namane A, Martinie B, Labadie JC. 2002. A proteomic study of *Escherichia coli* O157:H7 NCTC 12900 cultivated in biofilm or in planktonic growth mode. *FEMS Microbiol Lett* **215:** (1) 7.
Ver Berkmoes NC, Hettich RL, Bruce BD, Nguyen R, Savage TL. 2002. One- and two-dimensional LC/MS/MS analysis of *Arabidopsis thaliana* proteome. *LC GC North Am* (Suppl) 10.
Ver Berkmoes NC, Bundy JL, Hauser L, Asano KG, Razumovskaya J, Larimer F, Hettich RL, Stephenson JL. 2002. Integrating "top-down" and "bottom-up" mass spectrometric approaches for proteomic analysis of *Shewanella oneidensis*. *J Proteome Res* **1:** (3) 239.
Wait R, Miller I, Eberini I, Cairoli F, Veronesi C, Battocchio M, Gemeiner M, Gianazza E. 2002. Strategies for proteomics with incompletely characterized genomes: The proteome of *Bos taurus* serum. *Electrophoresis* **23:** (19) 3418.
Wang CC, Huang RP, Sommer M, Lisoukov H, Huang RC, Lin Y, Miller T, Burke J. 2002. Array based multiplexed screening and quantitation of human cytokines and chemokines. *J Proteome Res* **1:** (4) 337.
Wilson KA, McManus MT, Gordon ME, Jordan TW. 2002. The proteomics of senescence in leaves of white clover *Trifolium repens* (L.). *Proteomics* **2:** (9) 1114.

### 12 Protein structural genomics

Abramov YM, Vasiliev AM, Khlebnikov VA, Vasilenko RN, Kulikova NL, Kosarev IV, Ishchenko AT, Gillespie JR, Millett IS, Fink AL *et al*. 2002. Structural and functional properties of *Yersinia pestis* Caf1 capsular antigen and their possible role in fulminant development of primary pneumonic plague. *J Proteome Res* **1:** (4) 307.
Downes AM, Richardson BJ. 2002. Relationships between genomic base content and distribution of mass in coded proteins. *J Mol Evol* **55:** (4) 476.
Doytchinova IA, Blythe MJ, Flower DR. 2002. Additive method for the prediction of protein-peptide binding affinity. Application to the MHC class I molecule HLA-A0201. *J Proteome Res* **1:** (3) 263.
Gough J. 2002. The SUPERFAMILY database in structural genomics. *Acta Crystallogr D-Biol Crystallogr* **58:** (11) 1897.
Kwanyuen P, Allina SM, Weissinger AK, Wilson RF. 2002. A new form of crystalline rubisco and the conversion to its common dodecahedral form. *J Proteome Res* **1:** (5) 471.
Robson B, Mordasini T, Curioni A. 2002. Studies in the assessment of folding quality for protein modeling and structure prediction. *J Proteome Res* **1:** (2) 115.
Rupp B, Segelke BW, Krupka HI, Lekin TP, Schafer J, Zemla A, Toppani D, Snell G, Earnest T. 2002. The TB structural genomics consortium crystallization facility: Towards automation from protein to electron density. *Acta Crystallogr D-Biol Crystallogr* **58:** (10) 1514.
Uversky VN, Permyakov SE, Zagranichny VE, Rodionov IL, Fink AL, Cherskaya AM, Wasserman LA, Permyakov EA. 2002. Effect of zinc and temperature on the conformation of the $\gamma$ subunit of retinal phosphodiesterase: A natively unfolded protein. *J Proteome Res* **1:** (2) 149.

### 13 Metabolomics

Gavaghan CL, Wilson ID, Nicholson JK. 2002. Physiological variation in metabolic phenotyping and functional genomic studies: Use of orthogonal signal correction and PLS-DA. *FEBS Lett* **530:** (1-3) 191.
Kell DB. 2002. Metabolomics and machine learning: Explanatory analysis of complex metabolome data using genetic programming to produce simple, robust rules. *Mol Biol Rep* **29:** (1-2) 237.
Lin J, Qian J, Greenbaum D, Bertone P, Das R, Echols N, Senes A, Stenger B, Gerstein M. 2002. GeneCensus: Genome comparisons in terms of metabolic pathway activity and protein family sharing. *Nucleic Acids Res* **30:** (20) 4574.

### 14 Genomic approaches to development

Chen ZY, Corey DP. 2002. Understanding inner ear development with

Copyright © 2003 John Wiley & Sons, Ltd.

gene expression profiling. *J Neurobiol* **53:** (2) 276.
Lian Z, Kluger Y, Greenbaum DS, Tuck D, Gerstein M, Berliner N, Weissman SM, Newburger PE. 2002. Genomic and proteomic analysis of the myeloid differentiation program: Global analysis of gene expression during induced differentiation in the MPRO cell line. *Blood* **100:** (9) 3209.

### 15 Technological advances

Berger SJ, Lee SW, Anderson GA, Pasa-Tolic L, Tolic N, Shen YF, Zhao R, Smith RD. 2002. High-throughput global peptide proteomic analysis by combining stable isotope amino acid labeling and data-dependent multiplexed-MS/MS. *Anal Chem* **74:** (19) 4994.
Blonder J, Goshe MB, Moore RJ, Pasa-Tolic L, Masselon CD, Lipton MS, Smith RD. 2002. Enrichment of integral membrane proteins for proteomic analysis using liquid chromatography-tandem mass spectrometry. *J Proteome Res* **1:** (4) 351.
Chelius D, Bondarenko PV. 2002. Quantitative profiling of proteins in complex mixtures using liquid chromatography and mass spectrometry. *J Proteome Res* **1:** (4) 317.
Chen JZ, Lee CS, Shen YF, Smith RD, Baehrecke EH. 2002. Integration of capillary isoelectric focusing with capillary reversed-phase liquid chromatography for two-dimensional proteomics separation. *Electrophoresis* **23:** (18) 3143.
Delinsky DC, Greis KD. 2002. Capillary chromatography-coupled mass spectrometry with column switching for rapid identification of proteins from 2-dimensional electrophoresis gels. *J Proteome Res* **1:** (3) 279.
Dunmire V, Wu CL, Symmans WF, Zhang W. 2002. Increased yield of total RNA from fine-needle aspirates for use in expression microarray analysis. *Biotechniques* **33:** (4) 890.
El Atifi M, Dupre I, Rostaing B, Chambaz EM, Benabid AL, Berger F. 2002. Long oligonucleotide arrays on nylon for large-scale gene expression analysis. *Biotechniques* **33:** (3) 612.
Farbrother P, Muller S, Noegel AA, Eichinger L. 2002. Comparison of probe preparation methods for DNA microarrays. *Biotechniques* **33:** (4) 884.
Feldman AL, Costouros NG, Wang E, Qian M, Marincola FM, Alexander HR, Libutti SK. 2002. Advantages of mRNA amplification for microarray analysis. *Biotechniques* **33:** (4) 906.
Flora JW, Null AP, Muddiman DC. 2002. Dual-micro-ESI source for precise mass determination on a quadrupole time-of-flight mass spectrometer for genomic and proteomic applications. *Anal Bioanal Chem* **373:** (7) 538.
Geoghegan KF, Hoth LR, Tan DH, Borzillerl KA, Withka JM, Boyd JG. 2002. Cyclization of N-terminal *S*-carbamoylmethylcysteine causing loss of 17 Da from peptides and extra peaks in peptide maps. *J Proteome Res* **1:** (2) 181.
Gururaja T, Li WQ, Bernstein J, Payan DG, Anderson DC. 2002. Use of MEDUSA-based data analysis and capillary HPLC-ion-trap mass spectrometry to examine complex immunoaffinity extracts of RbAp48. *J Proteome Res* **1:** (3) 253.
Guzzetta AW, Thakur RA, Mylchreest IC. 2002. A robust micro-electrospray ionization technique for high-throughput liquid chromatography/mass spectrometry proteomics using a sanded metal needle as an emitter. *Rapid Commun Mass Spectrom* **16:** (21) 2067.
Gygi SP, Rist B, Griffin TJ, Eng J, Aebersold R. 2002. Proteome analysis of low-abundance proteins using multidimensional chromatography and isotope-coded affinity tags. *J Proteome Res* **1:** (1) 47.
He Y, Yeung ES. 2002. Rapid determination of protein molecular weight by the Ferguson method and multiplexed capillary electrophoresis. *J Proteome Res* **1:** (3) 273.
Johnson JR, Meng FY, Forbes AJ, Cargile BJ, Kelleher NL. 2002. Fourier-transform mass spectrometry for automated fragmentation and identification of 5-20 kDa proteins in mixtures. *Electrophoresis* **23:** (18) 3217.
Kasif S, Weng ZP, Derti A, Beigel R, DeLisi C. 2002. A computational framework for optimal masking in the synthesis of oligonucleotide microarrays - Online art. no. e106. *Nucleic Acids Res* **30:** (20) e106.
Klein J, Harding G, Klein E. 2002. A new isoelectric focusing gel for two-dimensional electrophoresis constructed in microporous hollow fiber membranes. *J Proteome Res* **1:** (1) 41.
Korencic D, Soll D, Ambrogelly A. 2002. A one-step method for in vitro production of tRNA transcripts: Online art. no. e105. *Nucleic Acids Res* **30:** (20) e105.
Larsen MR, Cordwell SJ, Roepstorff P. 2002. Graphite powder as an alternative or supplement to reversed-phase material for desalting and concentration of peptide mixtures prior to matrix-assisted laser desorption/ionization-mass spectrometry. *Proteomics* **2:** (9) 1277.
Li J, Pankratz M, Johnson JA. 2002. Differential gene expression patterns revealed by oligonucleotide *versus* long cDNA arrays. *Toxicol Sci* **69:** (2) 383.
Liu PR, Regnier FE. 2002. An isotope coding strategy for proteomics involving both amine and carboxyl group labeling. *J Proteome Res* **1:** (5) 443.
Lo LC, Pang TL, Kuo CH, Chiang YL, Wang HY, Lin JJ. 2002. Design and synthesis of class-selective activity probes for protein tyrosine phosphatases. *J Proteome Res* **1:** (1) 35.
Locke VL, Gibson TS, Thomas TM, Corthals GL, Rylatt DB. 2002. Gradiflow as a prefractionation tool for two-dimensional electrophoresis. *Proteomics* **2:** (9) 1254.
Mathesius U, Imin N, Chen HC, Djordjevic MA, Weinman JJ, Natera SHA, Morris AC, Kerim T, Paul S, Menzel C, Weiller GF, Rolfe BG. 2002. Evaluation of proteome reference maps for cross-species identification of proteins by peptide mass fingerprinting. *Proteomics* **2:** (9) 1288.
Mohan D, Lee CS. 2002. On-line coupling of capillary isoelectric focusing with transient isotachophoresis-zone electrophoresis: A two-dimensional separation system for proteomics. *Electrophoresis* **23:** (18) 3160.
Natsume T, Yamauchi Y, Nakayama H, Shinkawa T, Yanagida M, Takahashi N, Isobe T. 2002. A direct nanoflow liquid chromatography-tandem system for interaction proteomics. *Anal Chem* **74:** (18) 4725.
Natsume T, Taoka M, Manki H, Kume S, Isobe T, Mikoshiba K. 2002. Rapid analysis of protein interactions: On-chip micropurification of recombinant protein expressed in *Escherichia coli*. *Proteomics* **2:** (9) 1247.
Neususs C, Pelzing M, Macht M. 2002. A robust approach for the analysis of peptides in the low femtomole range by capillary electrophoresis-tandem mass spectrometry. *Electrophoresis* **23:** (18) 3149.
Nielsen ML, Bennett KL, Larsen B, Moniatte M, Mann M. 2002. Peptide and sequencing by orthogonal MALDI tandem mass spectrometry. *J Proteome Res* **1:** (1) 63.
Nuwaysir EF, Huang W, Albert TJ, Singh J, Nuwaysir K, Pitas A, Richmond T, Gorski T, Berg JP, Ballin J *et al*. 2002. Gene expression analysis using oligonucleotide arrays produced by maskless photolithography. *Genome Res* **12:** (11) 1749.
Pasa-Tolic L, Harkewicz R, Anderson GA, Tolic N, Shen YF, Zhao R, Thrall B, Masselon C, Smith RD. 2002. Increased proteome coverage for quantitative peptide abundance measurements based upon high performance separations and DREAMS FTICR mass spectrometry. *J Am Soc Mass Spectrom* **13:** (8) 954.
Pramanik BN, Mirza UA, Ing YH, Liu YH, Bartner PL, Weber PC, Bose MK. 2002. Microwave-enhanced enzyme reaction for protein mapping by mass spectrometry: A new approach to protein digestion in minutes. *Protein Sci* **11:** (11) 2676.
Puskas LG, Zvara A, Hackler L, Micsik T, Van Hummelen P. 2002. Production of bulk amounts of universal RNA for DNA microarrays. *Biotechniques* **33:** (4) 898.
Raffelsberger W, Dembele D, Neubauer MG, Gottardis MM, Gronemeyer H. 2002. Quality indicators increase the reliability of microarray data. *Genomics* **80:** (4) 385.
Randic M, Novic M, Vracko M. 2002. On characterization of dose variations of 2-D proteomics maps by matrix invariants. *J Proteome Res* **1:** (3) 217.
Rejtar T, Hu P, Juhasz P, Campbell JM, Vestal ML, Preisler J, Karger BL. 2002. Off-line coupling of high-resolution capillary electrophoresis to MALDI-TOF and TOF/TOF MS. *J Proteome Res* **1:** (2) 171.
Richter A, Schwager C, Hentze S, Ansorge W, Hentze MW, Muckenthaler M. 2002. Comparison of fluorescent tag DNA labeling methods used for expression analysis by DNA microarrays. *Biotechniques* **33:** (3) 620.
Ruotolo BT, Verbeck GF, Thomson LM, Woods AS, Gillig KJ, Russell DH. 2002. Distinguishing between phosphorylated and nonphosphorylated peptides with ion mobility-mass spectrometry. *J Proteome Res* **1:** (4) 303.
Sadygov RG, Eng J, Durr E, Saraf A, McDonald H, MacCross MJ,

Yates JR. 2002. Code developments to improve the efficiency of automated MS/MS spectra interpretation. *J Proteome Res* **1:** (3) 211.

Saluz HP, Iqbal J, Limmon GV, Ruryk A, Wu ZH. 2002. Fundamentals of DNA-chip/array technology for comparative gene-expression analysis. *Curr Sci* **83:** (7) 829.

Sawasaki T, Ogasawara T, Morishita R, Endo Y. 2002. A cell-free protein synthesis system for high-throughput proteomics. *Proc Natl Acad Sci U S A* **99:** (23) 14652.

Sommer AP, Franke RP. 2002. Near-field optical analysis of living cells *in vitro*. *J Proteome Res* **1:** (2) 111.

Spruill SE, Lu J, Hardy S, Weir B. 2002. Assessing sources of variability in microarray gene expression data. *Biotechniques* **33:** (4) 916.

Stadler F, Hales D. 2002. Highly-resolving two-dimensional electrophoresis for the study of insect proteins. *Proteomics* **2:** (9) 1347.

Sterrenburg E, Turk R, Boer JM, Van Ommen GB, Den Dunnen JT. 2002. A common reference for cDNA microarray hybridizations: Online art. no. e116. *Nucleic Acids Res* **30:** (21) e116.

Tu Y, Stolovitzky G, Klein U. 2002. Quantitative noise analysis for gene expression microarray experiments. *Proc Natl Acad Sci U S A* **99:** (22) 14031.

Wall DB, Berger SJ, Finch JW, Cohen SA, Richardson K, Chapman R, Drabble D, Brown J, Gostick D. 2002. Continuous sample deposition from reversed-phase liquid chromatography to tracks on a matrix-assisted laser desorption/ionization precoated target for the analysis of protein digests. *Electrophoresis* **23:** (18) 3193.

Wang HX, Kachman MT, Schwartz DR, Cho KR, Lubman DM. 2002. Continuous sample deposition from reversed-phase liquid chromatography to tracks on a matrix-assisted laser desorption/ionization precoated target for the analysis of protein digests. *Electrophoresis* **23:** (18) 3168.

Weidenhammer EM, Kahl BF, Wang L, Wang L, Duhon M, Jackson JA, Slater M, Xu X. 2002. Multiplexed, targeted gene expression profiling and genetic analysis on electronic microarrays. *Clin Chem* **48:** (11) 1873.

Weinberger SR, Viner RI, Ho P. 2002. Tagless extraction-retentate chromatography: A new global protein digestion strategy for monitoring differential protein expression. *Electrophoresis* **23:** (18) 3182.

Wetterhall M, Palmblad M, Hakansson P, Markides KE, Bergquist J. 2002. Rapid analysis of tryptically digested cerebrospinal fluid using capillary electrophoresis-electrospray ionization-Fourier transform ion cyclotron resonance-mass spectrometry. *J Proteome Res* **1:** (4) 361.

Yamagata A, Kristensen DB, Takeda Y, Miyamoto Y, Okada K, Inamatsu M, Yoshizato K. 2002. Mapping of phosphorylated proteins on two-dimensional polyacrylamide gels using protein phosphatase. *Proteomics* **2:** (9) 1267.

Yi EC, Marelli M, Lee H, Purvine SO, Aebersold R, Aitchison JD, Goodlett DR. 2002. Approaching complete peroxisome characterization by gas-phase fractionation. *Electrophoresis* **23:** (18) 3205.

Zhang B, Ma WL, Shi R, Li L, Guo QY, Zheng WL. 2002. Re-use of a stripped cDNA microarray. *Br J Biomed Sci* **59:** (2) 118.

Zhang RJ, Regnier FE. 2002. Minimizing resolution of isotopically coded peptides in comparative proteomics. *J Proteome Res* **1:** (2) 139.

Zhu H, Pan S, Gu S, Bradbury EM, Chen X. 2002. Amino acid residue specific stable isotope labeling for quantitative proteomics. *Rapid Commun Mass Spectrom* **16:** (22) 2115.

### 16 Bioinformatics

Arques DG, Lacan M, Michel CJ. 2002. Identification of protein coding genes in genomes with statistical functions based on the circular code. *Biosystems* **66:** (1-2) 73.

Backovic M, Gettins PGW. 2002. Insight into residues critical for antithrombin function from analysis of an expanded database of sequences that includes frog, turtle, and ostrich antithrombins. *J Proteome Res* **1:** (4) 367.

Bakay M, Zhao P, Chen J, Hoffman EP. 2002. A web-accessible complete transcriptome of normal human and DMD muscle. *Neuromuscular Disord* **12:** (Suppl 1) S125.

Bard JBL. 2002. Using bioinformatics to identify kidney genes. *Nephrol Dial Transplant* **17:** (S9) 62.

Berezikov E, Plasterk RHA, Cuppen E. 2002. GENOTRACE: cDNA-based local GENOme assembly from TRACE archives. *Bioinformatics* **18:** (10) 1396.

Brody JP, Williams BA, Wold BJ, Quake SR. 2002. Significance and statistical errors in the analysis of DNA microarray data. *Proc Natl Acad Sci U S A* **99:** (20) 12975.

Cho SY, Park KS, Shim JE, Kwon MS, Joo KH, Lee WS, Chang J, Kim H, Chung HC, Kim HO *et al*. 2002. An integrated proteome database for two-dimensional electrophoresis data analysis and laboratory information management system. *Proteomics* **2:** (9) 1104.

Curtis RK, Brand MD. 2002. Control analysis of DNA microarray expression data. *Mol Biol Rep* **29:** (1-2) 67.

Eddes JS, Kapp EA, Frecklington DF, Connolly LM, Layton MJ, Moritz RL, Simpson RJ. 2002. CHOMPER: A bioinformatic tool for rapid validation of tandem mass spectrometry search results associated with high-throughput proteomic strategies. *Proteomics* **2:** (9) 1097.

Geer LY, Domrachev M, Lipman DJ, Bryant SH. 2002. CDART: Protein homology by domain architecture. *Genome Res* **12:** (10) 1619.

Gibbons FD, Roth FP. 2002. Judging the quality of gene expression-based clustering methods using gene annotation. *Genome Res* **12:** (10) 1574.

Howe KL, Chothia T, Durbin R. 2002. GAZE: A generic framework for the integration of gene-prediction data by dynamic programming. *Genome Res* **12:** (9) 1418.

Issac B, Raghava GPS. 2002. GWFASTA: Server for FASTA search in eukaryotic and microbial genomes. *Biotechniques* **33:** (3) 548.

Kaderali L, Schliep A. 2002. Selecting signature oligonucleotides to identify organisms using DNA arrays. *Bioinformatics* **18:** (10) 1340.

Kim H, Zhao B, Snesrud EC, Haas BJ, Town CD, Quackenbush J. 2002. Use of RNA and genomic DNA references for inferred comparisons in DNA microarray analyses. *Biotechniques* **33:** (4) 924.

Li Y, Campbell C, Tipping M. 2002. Bayesian automatic relevance determination algorithms for classifying gene expression data. *Bioinformatics* **18:** (10) 1332.

Liu AY, Zhang Y, Gehan E, Clarke R. 2002. Block principal component analysis with application to gene microarray data classification. *Stat Med* **21:** (22) 3465.

Malmstrom L, Malmstrom J, Marko-Varga G, Westergren-Thorsson G. 2002. Proteomic 2DE database for spot selection, automated annotation, and data analysis. *J Proteome Res* **1:** (2) 135.

Mateos A, Dopazo J, Jansen R, Tu YH, Gerstein M, Stolovitzky G. 2002. Systematic learning of gene functional classes from DNA array expression data by using multilayer perceptrons. *Genome Res* **12:** (11) 1703.

Peterson LE. 2002. Factor analysis of cluster-specific gene expression levels from cDNA microarrays. *Comput Methods Program Biomed* **69:** (3) 179.

Pybus OG, Rambaut A. 2002. GENIE: Estimating demographic history from molecular phylogenies. *Bioinformatics* **18:** (10) 1404.

Randic M. 2002. A graph theoretical characterization of proteomics maps. *Int J Quantum Chem* **90:** (2) 848.

Randic M, Basak SC. 2002. A comparative study of proteomics maps using graph theoretical biodescriptors. *J Chem Inform Comput Sci* **42:** (5) 983.

Raychaudhuri S, Schutze H, Altman RB. 2002. Using text analysis to identify functionally coherent gene groups. *Genome Res* **12:** (10) 1582.

Sankoff D. 2002. Short inversions and conserved gene cluster. *Bioinformatics* **18:** (10) 1305.

Sillanpaa MJ. 2002. Mathematics-assisted mapping in analysis of medical disease. *Ann Med* **34:** (4) 291.

Stein LD, Mungall C, Shu SQ, Caudy M, Mangone M, Day A, Nickerson E, Stajich JE, Harris TW, Arva A *et al*. 2002. The Generic Genome Browser: A building block for a model organism system database. *Genome Res* **12:** (10) 1599.

Tabb DL, McDonald WH, Yates JR. 2002. DTASelect and contrast: Tools for assembling and comparing protein identifications from shotgun proteomics. *J Proteome Res* **1:** (1) 21.

Vu TT, Vohradsky J. 2002. Genexp: A genetic network simulation environment. *Bioinformatics* **18:** (10) 1400.

Wilkinson MD, Block D, Crosby WL. 2002. Genquire: Genome annotation browser/editor. *Bioinformatics* **18:** (10) 1398.

Xu HQ, Wu PR, Wu CFJ, Tidwell C, Wang YX. 2002. A smooth response surface algorithm for constructing a gene regulatory network. *Physiol Genomics* **11:** (1) 11.

Copyright © 2003 John Wiley & Sons, Ltd.
